# A reaction-diffusion model to understand granulomas formation inside secondary lobule during tuberculosis infection

**DOI:** 10.1371/journal.pone.0239289

**Published:** 2020-09-16

**Authors:** Martí Català, Clara Prats, Daniel López, Pere-Joan Cardona, Sergio Alonso

**Affiliations:** 1 Department of Physics, Universitat Politècnica de Catalunya, Barcelona, Catalonia, Spain; 2 Comparative Medicine and Bioimage Centre of Catalonia (CMCiB), Fundació Institut d’Investigació en Ciències de la Salut Germans Trias i Pujol. Badalona, Catalonia, Spain; 3 Experimental Tuberculosis Unit (UTE), Fundació Institut Germans Trias i Pujol (IGTP), Universitat Autònoma de Barcelona (UAB), Badalona, Catalonia, Spain; 4 Centro de Investigación Biomédica en Red de Enfermedades Respiratorias (CIBERES), Madrid, Spain; The University of Georgia, UNITED STATES

## Abstract

*Mycobacterium tuberculosis* (*Mtb*) is the causative agent for tuberculosis, the most extended infectious disease around the world. When Mtb enters inside the pulmonary alveolus it is rapidly phagocytosed by the alveolar macrophage. Although this controls the majority of inhaled microorganisms, in this case, Mtb survives inside the macrophage and multiplies. A posterior chemokine and cytokine cascade generated by the irruption of monocytes, neutrophils and posteriorly, by T-cells, does not necessarily stop the growth of the granuloma. Interestingly, the encapsulation process built by fibroblasts is able to surround the lesion and stop its growing. The success of this last process determines if the host enters in an asymptomatic latent state or continues into a life-threatening and infective active tuberculosis disease (TB). Understanding such dichotomic process is challenging, and computational modeling can bring new ideas. Thus, we have modeled the different stages of the infection, first in a single alveolus (a sac with a radius of 0.15 millimeters) and, second, inside a secondary lobule (a compartment of the lungs of around 3 cm^3^). We have employed stochastic reaction-diffusion equations to model the interactions among the cells and the diffusive transport to neighboring alveolus. The whole set of equations have successfully described the encapsulation process and determine that the size of the lesions depends on its position on the secondary lobule. We conclude that size and shape of the secondary lobule are the relevant variables to control the lesions, and, therefore, to avoid the evolution towards TB development. As lesions appear near to interlobular connective tissue they are easily controlled and their growth is drastically stopped, in this sense secondary lobules with a more flattened shape could control better the lesion.

## Introduction

Tuberculosis (TB) is an infectious disease that on 2017 killed 1.6 million people [1]. The same year, nearly 10 million people developed the disease. Despite our best efforts, *Mycobacterium tuberculosis* (*Mtb*) remains the bacteria able to cause the highest mortality by itself. Since World Health Organization declaration of TB public health emergency in 1994 [2], TB death rate has been reduced from 23% in 2000 to 16% nowadays [1]. In 2015, the TB End Strategy stated the goal of reducing its incidence by 50% in 2025 and 90% in 2035, as well as of reducing TB death to 75% in 2025 and 95% in 2035 [3]. It is estimated that between one quarter and one third of world population is infected with *Mtb* and that around a 10% of them will develop an active TB disease in the future years.

Tuberculosis natural history has an extraordinary complexity and, in fact, there are still too many unknowns [4]. Tuberculosis infection starts when an *Mtb* is phagocyted by an alveolar macrophage (AM) at a pulmonary alveolus. *Mtb* resists bactericidal mechanisms induced by AM and replicates inside [5]. This capacity is the biggest challenge of *Mtb* and the main reason of its high mortality in humans along history. Under proper conditions, *Mtb* replicates approximately once a day [6]. When the intracellular bacterial load overcomes AM maximum tolerability, macrophage necrosis is triggered and thereby bacilli enter the extracellular milieu. These bacilli are phagocyted by other AM, which also fail to control the bacillary growth and are likewise destroyed. This cycle is followed by a local inflammatory response and potentially ends once the specific immune response appears and controls it. This is the end of the progressive infection, which finally leaves an encapsulated TB lesion [6, 7]. Fibroblasts cells lead encapsulation with collagen, fibrin and other molecules. Fibroblasts cells are mainly located at pulmonary membranes like intralobular *septae*. In fact, this suggests that lungs structure may have an important role in TB infection dynamics, since initial infection distance to the nearest pulmonary membrane may determine the encapsulation capacity and, therefore, the final lesions size and bacillary load. Lungs are composed by around 2500 secondary lobules, and each lobule has an approximate volume of 3 cm^3^.

There are two types of lesions: proliferative and exudative lesions. Proliferative lesions are mainly based in macrophages and lymphocytes, with intracellular bacilli inside the macrophages and minimal necrosis, controled through time; exudative lesions are mainly based on necrotic tissue caused by neutrophilic infiltration where a lot of extracellular bacilli are accumulated [8–10].

If the response of the host is adequate, an *Mtb* infection can be completely cleared from the organism [11] or it can enter an asymptomatic latent state where the host is infected but not sick and cannot infect other people, corresponding to latent tuberculosis infection (LTBI). Nevertheless, if the immune and the inflammatory responses are not correctly balanced, the host can develop a more compromised situation, the active tuberculosis disease (ATB).

Systems biology and computational models are great tools for increasing TB understanding [12]. Last years, several TB models have been built for a better understanding of different processes related with TB natural history [4]. In particular, GranSim [13] is a hybrid model that, under several modifications, is able to reproduce granulomas formation [14], encapsulation [15] and study drug resistance [16], among others. Bubble model [17, 18] is an Agent-based Model that reproduces the formation and growth of lesions, as well as the coalescence of neighboring lesions and their spreading by means of bronchial endogenous reinfection processes. Other mathematical models study different states in macrophage-bacilli competitivity [19].

In this work we develop a mathematical model to reproduce *Mtb* infection dynamics in an alveolus by means of Stochastic Differential Equations (SDEs). It is then generalized to a three-dimensional stochastic reaction-diffusion model that incorporates the space to reproduce *Mtb* infection, lesions formation and encapsulation in a secondary lobule. Finally, we identify the factors that determine lesions size and encapsulation time.

## Materials and methods

### Model structure

Two different approaches are considered: the modeling of a single alveolus and the modeling of a secondary lobule. The difference between both models is the dimensionality of the space where the simulation takes place. Single alveolus system is a non dimensional model, therefore modeled by ordinary differential equations, and the secondary lobule is a three dimensional system modeled by partial differential equations.

**Fig 1:**
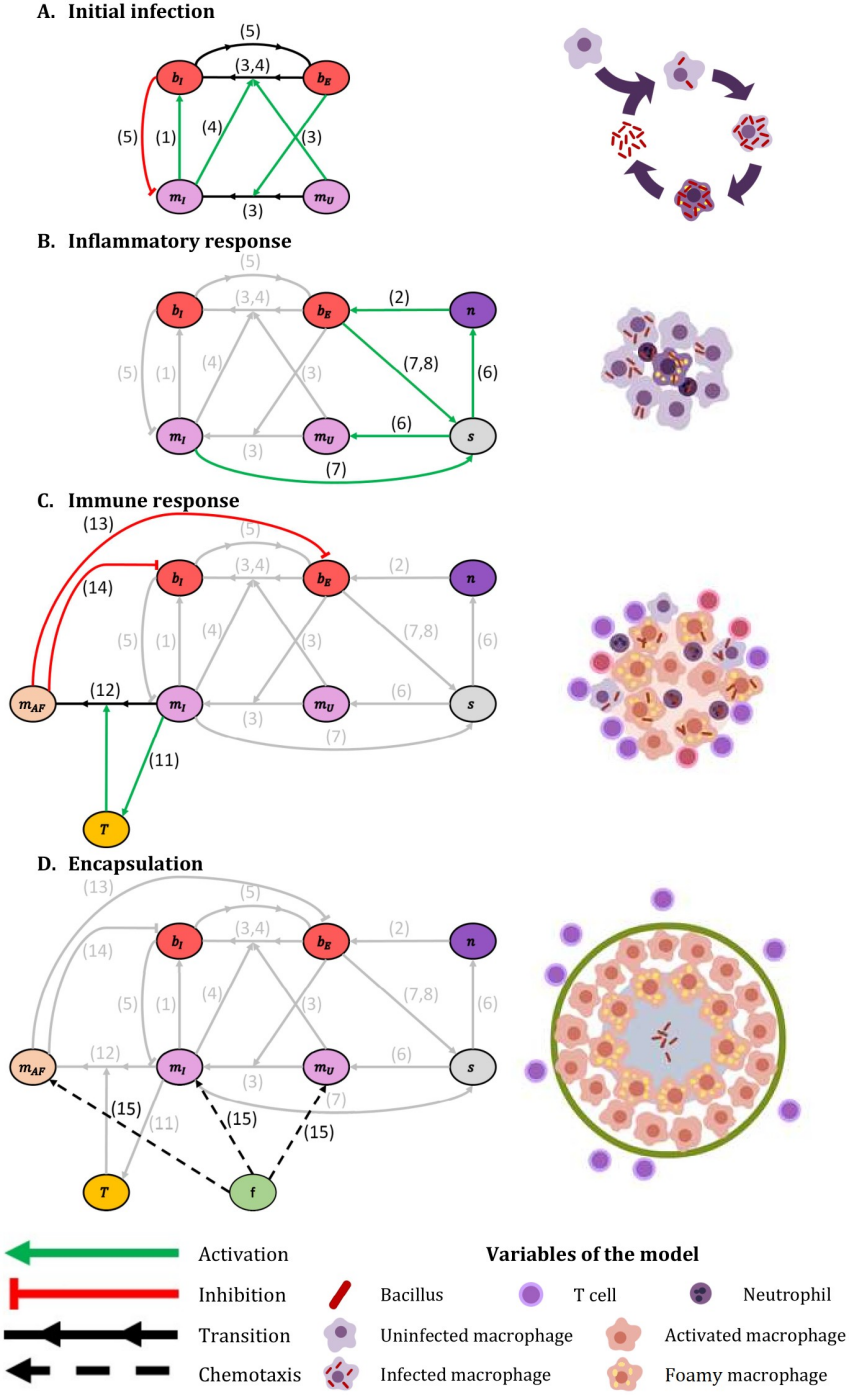
Four principal model’s modules. Interaction between different model elements. (A) Initial infection: interaction between bacilli and macrophages. (B) Inflammatory response: triggered by the presence of extracellular bacilli. (C) Immune response: infected macrophages trigger the mechanisms that activate this response. (D) Encapsulation: led by fibroblasts, it takes place when a lesion makes contact with a pulmonary membrane. Green arrows: activation; red arrows: inhibition; black arrows: transition; dotted arrows: chemo-taxis. Numbers identify specific processes: 1 intracellular bacilli growth, 2 extracellular bacilli growth, 3 phagocytosis by non-infected macrophages, 4 phagocytosis by infected macrophages, 5 macrophages’ lysis, 6 inflammatory response entrance, 7 inflammatory response activation, 8 inflammatory response inhibition, 11 immune response entrance, 12 macrophages activation, 13 extracellular bacilli elimination, 14 intracellular bacilli drainage and 15 fibroblasts chemo-taxis. See model’s details in Eqs (1)–(10). Part of this figure is adapted from [6].

Next we explain the interactions among the different cells and elements of the model. We consider 5 types of cells in this model: bacilli (*b*), macrophages (*m*), neutrophils (*n*), T cells (*T*) and fibroblasts (*f*). Bacilli are distinguished between intracellular (*b_I_*), i.e. bacilli inside a macrophage, and extracellular (*b_E_*), i.e. bacilli in the extracellular milieu. Accordingly, macrophages can be uninfected (*m_U_*), i.e. they do not have bacilli inside, infected (*m_I_*), i.e. they present bacilli inside but are not able to eliminate them, or activated-foamy (*m_AF_*), i.e. they present bacilli inside, are able to eliminate extracellular bacilli (activated) and drain intracellular ones (foamy). Volume occupied by death elements (*V_O_*) and local inflammatory response (*s*) are also considered. Therefore, we consider the evolution of 8 types of cells and 2 additional processes. We take into account 16 processes, which lead to 10 reaction-diffusion equations, one for each element. These processes are described below. Note that the numbers in parentheses relate to the description with the corresponding terms in the model equations and with the processes depicted in Fig 1.

While the initial infection is closely related with the dynamics inside of an alveolus, the final part of the process, the encapsulation of the granuloma, takes into consideration the structure of the secondary lobule. For the first modeling appoach based on a single alvoulus, we study the effect of inflammatory and immune responses at the level of the alveolus, while for the second approach such responses are considered at a larger scale, the secondary lobule. For both approaches, we divide the whole evolution in four differential modules:

#### Initial infection

*Mtb* infection starts when an AM phagocytes a bacillus in the alveolar surface (3, *phagocytosis by non-infected macrophages*). In this process, the extracellular bacilli become intracellular and non-infected macrophage becomes infected. *Mtb* resist bactericidal mechanisms induced by AM and replicates inside it (1, *intracellular bacilli growth*), as they use infected macrophages as sustenance. When intracellular bacillary load overcomes macrophage maximum tolerability (*α*), macrophage necrosis is triggered and thereby bacilli enter the extracellular milieu (5, *macrophages’ lysis*). Intracellular bacilli released become extracellular and the infected macrophage is eliminated. Then, other AM from closer alveoli enter the infected alveolus and the bacilli growth-macrophage lysis process occurs again. Infected macrophages also phagocyte extracellular bacilli (4, *phagocytosis by infected macrophages*) so that these extracellular bacilli also become intracellular. An explanatory schematic of this module is shown in Fig 1A.

#### Inflammatory response entrance

Macrophages death and bacillary load’s increase trigger inflammatory response (7, *inflammatory response activation*). The variable *s* is increased from its initial value, 0, up to 1 after 3-5 days. This value is an approximation based on experimental observations [20]. When *s* = 1, inflammatory response is fully active. This increase in *s* leads to an entrance of neutrophils and uninfected macrophages in the alveolus (6, *inflammatory entrance*). The presence of neutrophils may be used by extracellular bacilli as a sustenance to grow (2, *extracellular bacilli growth*). There is a limit of how many extracellular bacilli can grow on a neutrophil (*δ*). If bacillary load decreases and infected macrophages disappear, inflammatory response is inhibited (8, *inflammatory response inhibition*). Macrophages and neutrophils cascade is unable to contain the infection, in fact, bacillary load grows exponentially. An explanatory scheme of this module is shown in Fig 1B.

#### Immune response

Some infected macrophages trigger dendritic cells that travel to Lymph node to trigger immune response. Immune response consists of a T cells flow to the affected alveoli (11, *immune response entrance*). Infected macrophages are activated by contact with T cells (12, *macrophages activation*). This activated macrophages are able to phagocyte extracellular bacilli and eliminate them (13, *extracellular bacilli elimination*). Activated macrophages have a short lifespan. When they are not able to eliminate more bacilli they convert to foamy macrophages [21–23]. These foamy macrophages leave the alveolus by draining the intracellular bacilli inside them (14, *intracellular bacilli drainage*). All the elements have a certain lifespan; when they die, they leave a corpse that occupies a fraction of the space (9, *death rate*). A scheme of this module is shown in Fig 1C. Even when the líving-time of the neutrophils is limited (between 5 and 135 hours [24]), as they made neutrophilic extracellular nets (NETs) [25], and become part of the necrotic tissue, we have extended significantly the time of degradation. An explanatory scheme of this module is shown in Fig 1C.

#### Encapsulation

During all these processes all elements diffuse, therefore occupying surrounding alveoli (16, *diffusion*). All elements diffuse by their own except intracellular bacilli, whose diffusion is conditioned by infected macrophages that contain them. The initial infection forms a lesion due to cell accumulation. When lesions size is big enough to get in contact with pulmonary lobule membranes (interlobular connective tissue), encapsulation process is triggered. Encapsulation is led by fibroblasts cells that chemo-tact macrophages gradient (15, *fibroblast chemo-taxis*). Initially, fibroblasts cells are located at pulmonary lobules membranes. An explanatory scheme of this module is shown in Fig 1D.

In Fig 1, a summary of the relations between the 10 variables of the model are shown, together with the different interactions involved, organized in the four modules previously mentioned. The corresponding 10 reaction-diffusion equations of the model are:

$$\begin{array}{c} \begin{array}{cc} \frac{d}{dt}b_{I}= & \underset{\left(1\right)}{\underset{︸}{\mu b_{I}(1-{\left(\frac{b_{I}}{\alpha m_{I}}\right)}^{3})}}+\underset{\left(3\right)}{\underset{︸}{\gamma gm_{U}b_{E}}}+\underset{\left(4\right)}{\underset{︸}{\gamma gm_{I}b_{E}(1-{\left(\frac{b_{I}}{\alpha m_{I}}\right)}^{3})}}\\ & -\underset{\left(5\right)}{\underset{︸}{\alpha (\beta b_{I}+\left(1-\beta \alpha \right)m_{I})}}-\underset{\left(9\right)}{\underset{︸}{\lambda_{b}b_{I}}}-\underset{\left(14\right)}{\underset{︸}{\frac{\alpha }{\tau_{m}}m_{AF}}}+\underset{\left(16\right)}{\underset{︸}{\nabla (gD_{m}\frac{b_{I}}{m_{I}}\nabla m_{I})}}, \end{array} \end{array}$$ (1)

$$\begin{array}{c} \begin{array}{cc} \frac{d}{dt}b_{E}= & \underset{\left(2\right)}{\underset{︸}{\mu b_{E}(1-{\left(\frac{b_{E}}{\delta n}\right)}^{3})}}-\underset{\left(3\right)}{\underset{︸}{\gamma gm_{U}b_{E}}}-\underset{\left(4\right)}{\underset{︸}{\gamma gm_{I}b_{E}(1-{\left(\frac{b_{I}}{\alpha m_{I}}\right)}^{3})}}\\ & +\underset{\left(5\right)}{\underset{︸}{\alpha (\beta b_{I}+\left(1-\beta \alpha \right)m_{I})}}-\underset{\left(9\right)}{\underset{︸}{\lambda_{b}b_{E}}}-\underset{\left(13\right)}{\underset{︸}{\nu m_{AF}g}}+\underset{\left(16\right)}{\underset{︸}{\nabla (gD_{b}\nabla b_{E})}}, \end{array} \end{array}$$ (2)

$$\begin{array}{c} \frac{d}{dt}m_{U}=-\underset{\left(3\right)}{\underset{︸}{\gamma gm_{U}b_{E}}}+\underset{\left(6\right)}{\underset{︸}{k_{1}(\frac{b_{E}}{m})sg}}-\underset{\left(9\right)}{\underset{︸}{\lambda_{m}m_{U}}}+\underset{\left(16\right)}{\underset{︸}{\nabla (gD_{m}\nabla m_{U})}}, \end{array}$$ (3)

$$\begin{array}{c} \frac{d}{dt}m_{I}=\underset{\left(3\right)}{\underset{︸}{\gamma gm_{U}b_{E}}}-\underset{\left(5\right)}{\underset{︸}{\left(\beta b_{I}+\left(1-\beta \alpha \right)m_{I}\right)}}-\underset{\left(12\right)}{\underset{︸}{\xi m_{I}Tg}}+\underset{\left(16\right)}{\underset{︸}{\nabla (gD_{m}\nabla m_{I})}}, \end{array}$$ (4)

$$\begin{array}{c} \frac{d}{dt}m_{AF}=\underset{\left(12\right)}{\underset{︸}{\xi m_{I}Tg}}-\underset{\left(14\right)}{\underset{︸}{\frac{1}{\tau_{m}}m_{AF}}}+\underset{\left(16\right)}{\underset{︸}{\nabla (gD_{m}\nabla m_{AF})}}, \end{array}$$ (5)

$$\begin{array}{c} \frac{d}{dt}n=\underset{\left(6\right)}{\underset{︸}{k_{2}(\frac{b_{E}}{m})sg}}-\underset{\left(10\right)}{\underset{︸}{\frac{1}{t_{n}}n}}+\underset{\left(16\right)}{\underset{︸}{\nabla (gD_{n}\nabla n)}}, \end{array}$$ (6)

$$\begin{array}{c} \frac{d}{dt}T=-\underset{\left(9\right)}{\underset{︸}{\lambda_{T}T}}+\underset{\left(11\right)}{\underset{︸}{\kappa m_{I}sg}}+\underset{\left(16\right)}{\underset{︸}{\nabla (gD_{T}\nabla T)}}, \end{array}$$ (7)

$$\begin{array}{c} \frac{d}{dt}f=\underset{\left(15\right)}{\underset{︸}{\nabla (g\chi_{f}|\nabla g|\nabla f)}}, \end{array}$$ (8)

$$\begin{array}{c} \frac{d}{dt}V_{O}=\underset{\left(5\right)}{\underset{︸}{\left(\beta b_{I}+\left(1-\beta \alpha \right)m_{I}\right)V_{m}}}+\underset{\left(9\right)}{\underset{︸}{\lambda_{m}m_{U}V_{m}}}+\underset{\left(9\right)}{\underset{︸}{\lambda_{T}TV_{T}}}+\underset{\left(10\right)}{\underset{︸}{\frac{1}{t_{n}}nV_{n}}}, \end{array}$$ (9)

$$\begin{array}{c} \frac{d}{dt}s=\underset{\left(7\right)}{\underset{︸}{\rho_{U}m_{I}b_{E}\left(1-s\right)}}-\underset{\left(8\right)}{\underset{︸}{\rho_{D}\frac{s}{b_{E}+1}}}; \end{array}$$ (10)

where g is the unoccupied fraction of volume in each alveolus:

$$\begin{array}{c} g=1-\frac{\left(m_{U}+m_{I}+m_{AF}\right)V_{m}+nV_{n}+TV_{T}+V_{O}}{V_{a}}, \end{array}$$ (11)

and *k*_1_(*x*) and *k*_2_(*x*) are the entrance rate of macrophages and neutrophils, respectively. They are quadratic functions that depend on extracellular bacilli—macrophages ratio. When this ratio is high enough, immune response is neutrophils-based (NBR) and most of the entering elements are neutrophils, otherwise, if macrophages are able to control extracellular bacilli, the immune response is macrophages-based (MBR) and most of the entering elements are macrophages. The threshold that differentiates both is half of macrophages tolerability (*α*/2). The total amount of external agents flux (*Υ*) is conserved:

$$\begin{array}{c} k_{1}\left(x\right)\cdot V_{m}+k_{2}\left(x\right)\cdot V_{n}=ϒ, \end{array}$$ (12)

$$\begin{array}{c} k_{1}\left(x\right)=\frac{ϒ}{V_{m}}\frac{4\epsilon^{+}x^{2}+\epsilon^{-}\alpha^{2}}{4x^{2}+\alpha^{2}}, \end{array}$$ (13)

$$\begin{array}{c} k_{2}\left(x\right)=\frac{ϒ}{V_{n}}\frac{4\left(1-\epsilon^{+}\right)x^{2}+\left(1-\epsilon^{-}\right)\alpha^{2}}{4x^{2}+\alpha^{2}}. \end{array}$$ (14)

**Fig 2:**
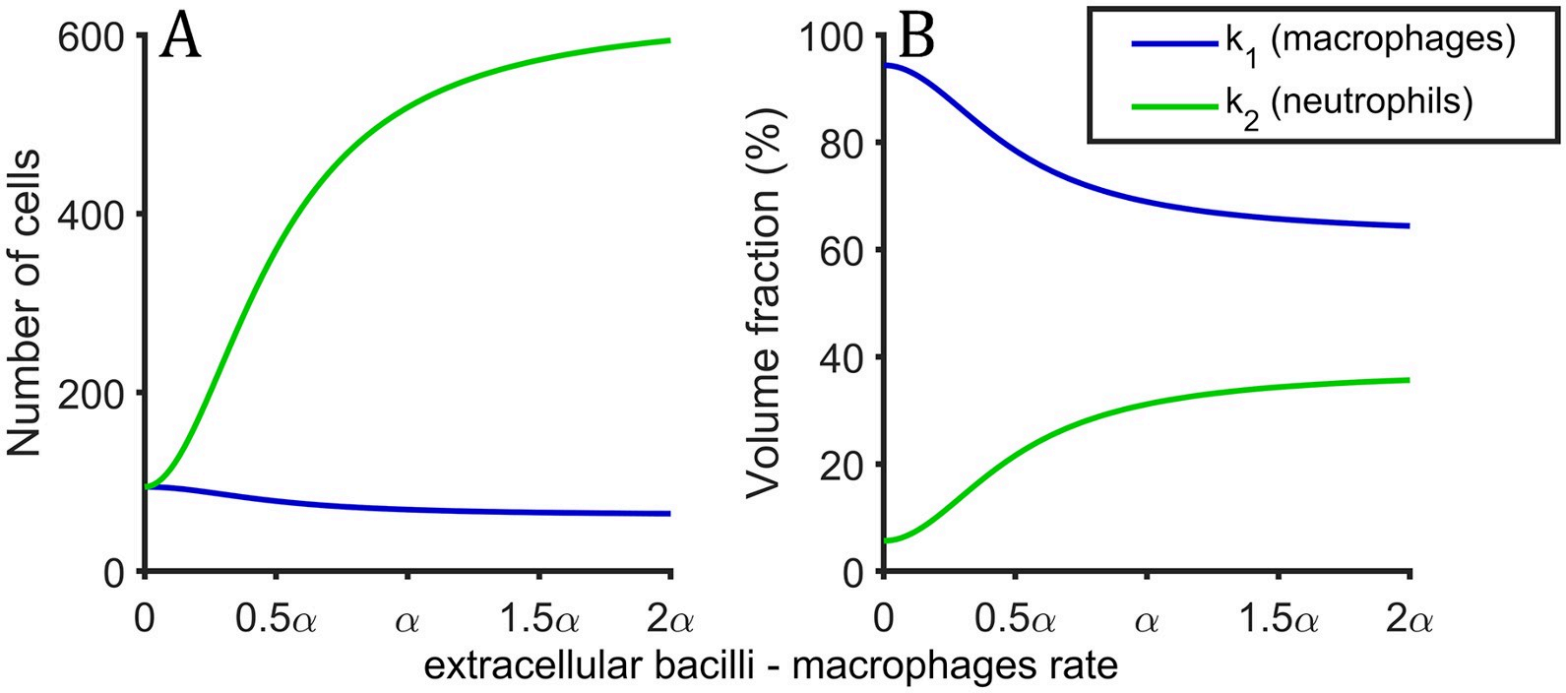
Inflammatory response profile. Profile of the inflammatory response as a function of extracellular bacilli and total amount of macrophages. (A) Number of cells that enter per day at the alveoli when inlfammatory response is fully activated. (B) Volume fraction of macrophages (blue) and neutrophils (green) that enter in alveoli due to inflammatory response.

Macrophages and neutrophils entrance rate profile per day can be observed in Fig 2 for values of extracellular bacilli and total amount of macrophages ratio between 0 and 2*α*.

Note that the 3D diffusion equations part is marked in blue. If the blue parts of the equation are not considered, we recover the equations for a single alveolus model.

### Parameters

This model depends on a total of 27 parameters that are obtained from bibliography or adjusted from experimental observations. All the parameters, their baseline value and corresponding references are shown in Table 1. Parameters *Υ*, *κ* and *ξ* are adjusted to reproduce Bru & Cardona observations [26]. Parameter *δ* is adjusted from Marzo et al figures [20]. Parameter *ρ* is adjusted from Marzo et al observations [20]. Parameters *β* and *γ* are computed using simple Agent-Based Models (ABM).

**Table 1:** Parameters description and values. Parameter with source ABM is adjusted from Agent-Based Model, details are on the text. Parameter with source ABM* is fitted from ABM and corrections are implemented. Parameters with adj. source were adjusted to reproduce tuberculosis infection dynamics according to that source. Parameters *D_b_*, *D_m_*, *D_n_*, *D_T_* and *χ_f_* are marked with ⋆ because they are not directly explored. These parameters are explored using D and DF parameters, related with Eq 24. Parameter D is explored between half and double its baseline value (4.6·10^-9^) and DF is explored between 0.5 and 4.

parameter	description	values	baseline value	units	explored values	source
*μ*	macrophages growing rate	0.69–1.04	0.693	day^-1^	0.35-1.39	[6, 27]
*α*	macrophages maximum tolerability	32–64	60	-	30-120	[6]
*β*	bacilli kill macrophages	0.01–0.04	0.0182	day^-1^	adj.	ABM
*γ*	phagocytation rate	8 ·10^-4^–8	0.05	day^-1^	0.025-0.1	ABM*
*δ*	neutrophils capacity	10-40	30	-	15-60	adj. [20]
*κ*	T cells recuitment		0.8	day^-1^	0.4-1.6	[19, 26]
*ξ*	macrophages activation		0.01	day^-1^	0.005-0.02	[19, 26]
*ν*	activated killing rate		150	day^-1^	75-300	adj. [6]
*ρ*_*U*_	inflammatory activation		10^-4^	day^-1^	10^-5^-10^-3^	adj. [20]
*ρ*_*D*_	inflammatory inhibition		1	day^-1^	no	adj. [20]
*Υ*	inflammatory flow		5 ⋅ 10^5^	*μ*m^3^day^-1^	2 ⋅ 10^5^-10^6^	adj. [6]
*ϵ*^+^	macrophages proportion in NBR		5/8	-	no	adj. [6]
*ϵ*^−^	macrophages proportion in MBR		50/53	-	no	[28]
λ_*b*_	bacilli death rate	1- 8 ⋅ 10^-3^	10^-2^	day^-1^	0.005-0.02	[29]
λ_*m*_	macrophages death rate		0.023	day^-1^	0.01-0.05	[30]
*τ*_*m*_	activated macrophages lifespan	3–10	5	day	2.5-10	[6, 31]
*t*_*n*_	neutrophils degradation time		100	day	50-200	adj.
λ_*T*_	T cells death rate	0.20–0.33	0.33	day^-1^	0.16-0.66	[6]
*V*_*m*_	macrophage volume		5000	*μ*m^3^	no	[32]
*V*_*n*_	neutrophil volume		300	*μ*m^3^	no	[33]
*V*_*T*_	T cell volume		200	*μ*m^3^	no	[34]
*V*_*a*_	alveolus volume	4 ·10^6^–6 ·10^7^	10^7^	*μ*m^3^	no	[35]
*D*_*b*_	bacilli diffusion	*D*_*m*_/10	4.6 ·10^-10^	cm^2^s^-1^	⋆	[31]
*D*_*m*_	macrophages diffusion	10^-5^–10^-11^	4.6 ·10^-9^	cm^2^s^-1^	⋆	[31]
*D*_*n*_	neutrophils diffusion	10^-5^–10^-11^	4.6 ·10^-9^	cm^2^s^-1^	⋆	[31]
*D*_*T*_	T cells diffusion	10^-5^–10^-11^	4.6 ·10^-9^	cm^2^s^-1^	⋆	[31]
*χ*_*f*_	fibroblasts chemotaxis coefficient		9.2 ·10^-9^	cm^2^s^-1^	⋆	adj. [6]

#### Adjusting *β*

The parameter *β* is fitted by means of an ad-hoc ABM designed for studying the distribution of *b_I_* among *m_I_* and the resulting amount of macrophages’ lyses. We develop a simple ABM where macrophages are the agents with only one property: the number of bacilli inside them. All other elements behave like the model described before. There is no diffusion, as just one alveolus is considered (single alveolus model). Macrophage lysis is triggered when its bacillary load is higher than macrophages maximum tolerability (*α*). Macrophages death rate due to bacilli growth is computed at each time step. As a result of the simulation, a function that relates the number of intracellular bacilli, number of infected macrophages and lysis rate due to intracellular bacilli growth can be fitted:

$$\begin{array}{c} \text{lysis}\left(m_{I},b_{I}\right)=\beta_{1}b_{I}+\beta_{2}m_{I}; \end{array}$$ (15)

where *β*_*i*_ are the adjusted constants. Fitting goodness R^2^ is computed and values over 0.8 are obtained for different parameters sets exploring diverse initial conditions. Using baseline values and a set of random initial conditions we obtain: *β*_1_ = 0.01816 day^-1^, *β*_2_ = −0.1007 day^-1^, R^2^ = 0.895. Note that, as expected, the number of bacilli needed to kill one macrophage is *α*:

$$\begin{array}{c} \beta_{1}\cdot \alpha +\beta_{2}\cdot 1\approx 1. \end{array}$$ (16)

Then, imposing Eq (16) and redefining *β*_1_ as *β* we obtain a new expression for the lysis function:

$$\begin{array}{c} \text{lysis}\left(m_{I},b_{I}\right)=\beta b_{I}+\left(1-\beta \alpha \right)m_{I}. \end{array}$$ (17)

Using baseline values we obtain *β* = 0.0182 day^−1^ and R^2^ = 0.884. The value of *β* is calculated at the start of every simulation and depend on the parameters set used. Parameters *α* and *μ* are the parameters that mostly determine *β* value.

#### Adjusting *γ*

The parameter *γ* is also estimated using another ad-hoc ABM that simulates a simple macrophages-bacilli interaction in an alveolus. We consider a 2D system where bacilli are considered fixed rectangles of width 0.6 *μ*m and length 4 *μ*m [36] and macrophages are mobile circles with radius r_m_ = 10.6 *μ*m [32]. Simulations take place in a L × L square with periodic boundary conditions which represent alveolar surface, L = 532 *μ*m [35]. Macrophages diffuse moving at constant velocity but with a changing direction. Using Fürth formula [37] a diffusive movement can be characterized by the following equations:

$$\begin{array}{c} \frac{d}{\text{dt}}\vec{x}\left(t\right)=\vec{v}\left(t\right), \end{array}$$ (18)

$$\begin{array}{c} \vec{v}\left(t\right)=v_{0}\cdot \left(\cos\theta \left(t\right),\sin\theta \left(t\right)\right), \end{array}$$ (19)

$$\begin{array}{c} \frac{d}{\text{dt}}\theta \left(t\right)=\sigma \cdot \eta \left(t\right); \end{array}$$ (20)

where *v*_0_ is a constant velocity that depends on diffusive coefficient, *D*_*m*_, and persistence time, *τ*_*p*_, that for macrophages can be approximated as *τ*_*p*_ = 2000 s [38]:

$$\begin{array}{c} v_{0}=\sqrt{\frac{2D_{m}}{\tau_{p}}}; \end{array}$$ (21)

and *η*(*t*) is white noise of amplitude *σ* that depends on persistence and time step, dt.

$$\begin{array}{c} \sigma =\sqrt{\frac{2}{\tau_{p}\text{dt}}}. \end{array}$$ (22)

At each time step, macrophages move and phagocyte bacilli at a distance smaller than its radius, (r_m_). These bacilli are eliminated from the system. Every time that a macrophage phagocytes a bacillus, macrophage is stopped during t_phag_ = 10 minutes [27]. At each time step, the number of bacilli, macrophages and phagocytations that take place are computed. Different random initial conditions are explored. As a result, the phagocyte ratio can be approximated as:

$$\begin{array}{c} \text{phagocyte}\left(m,b_{E}\right)=\gamma mb_{E}. \end{array}$$ (23)

Different sets of parameters adjust to different *γ* values. R^2^ is computed being always higher than 0.90. Using baseline values *γ* = 0.56 day^-1^ and R^2^ = 0.9882. This simplified model give us a range of values for *γ*, *γ*_min_ = 8 ⋅ 10^-4^ day^-1^ (*D*_*m*_ = 10^-11^ cm^2^ s^-1^) and *γ*_max_ = 7.6 day^-1^ (*D*_*m*_ = 10^-5^ cm^2^ s^-1^). Due to biological factors as alveolus saturation, long range interactions and occupation factor we considered *γ* smaller and its baseline value was set to *γ* = 0.05 day^-1^.

### Implementation

The model was implemented in MATLAB using Poisson *τ*-leap method to integrate differential equations [39], *τ* = 0.1 days for the one alveolus model and *τ* = 0.001 days for the secondary lobule model. Diffusion was computed with finite differences, Δ*x* = 0.3 mm. Transition probabilities were determined by the differential equation and the success or not was determined using Poisson random numbers to assure that all quantities are integers. Poisson random numbers are also used in finite differences.

For the secondary lobule model (3D simulations) we consider a cubic secondary lobule of 3.4 cm^3^ (1.5 × 1.5 × 1.5 cm). Secondary lobule is formed by 125000 (50 × 50 × 50) alveoli, cubes of 0.3 mm side [35]. Reaction equations take place in each alveoli and diffusion is considered with surrounding alveoli. The cube is surrounded by a thin membrane (1 alveolus thickness) where fibroblasts concentration is maximum. Membrane fibroblasts are considered inexhaustible (boundaries are always full of fibroblasts).

## Results

### Model of a single alveolus

#### Two final states are observed: Proliferative and exudative scenarios

**Fig 3:**
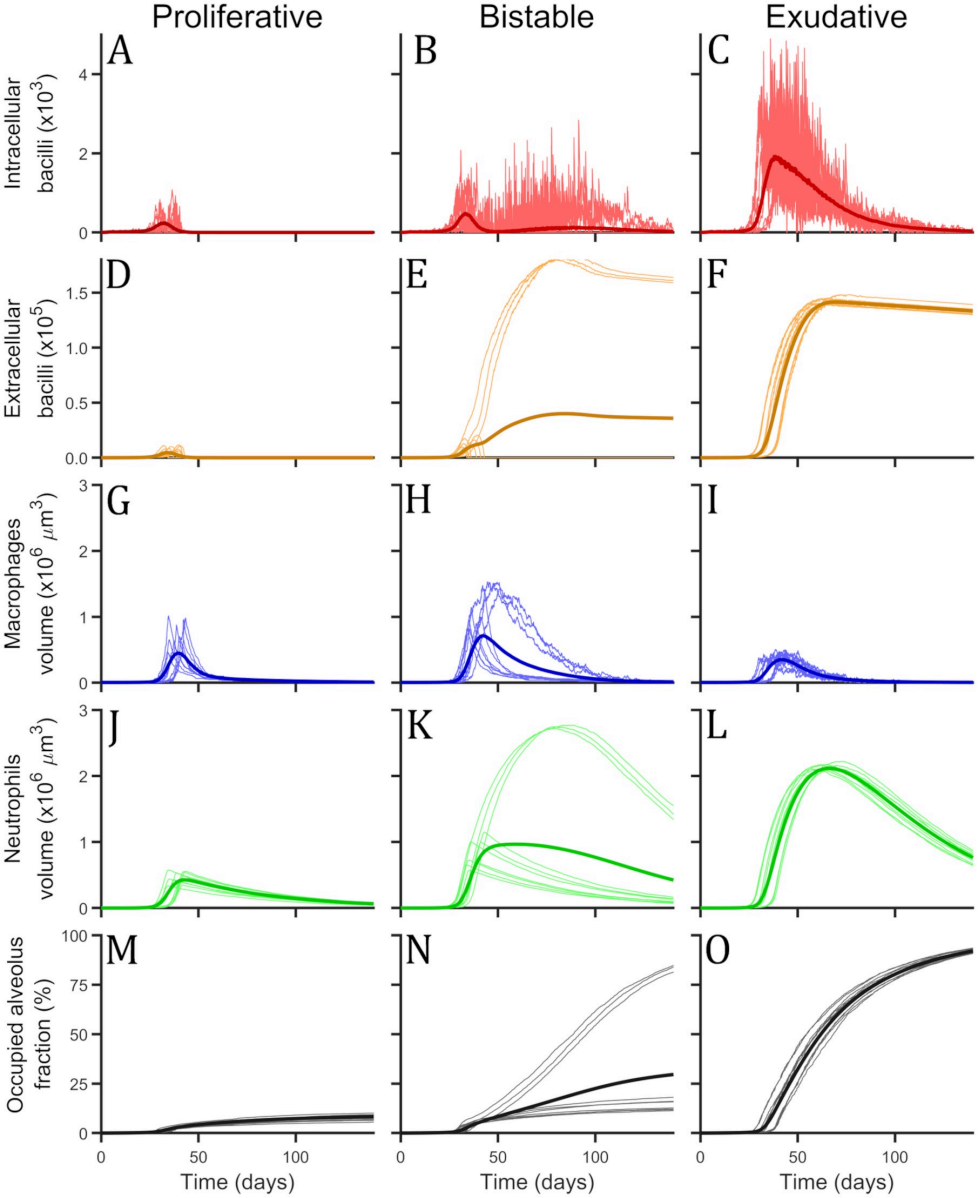
Single alveolus model simulations with three different set of parameters. (A, D, G, J, M) Proliferative set, (B, E, H, K, N) bistable set and (C, F, I, L, O) exudative set. For each set of parameters there can be seen the total amount of intracellular bacilli, in red (A, B, C), the total amount of extracellular bacilli, in orange (D, E, F), the total volume occupied by macrophages, in blue (G, H, I), the total volume occupied by neutrophils, in green (J, K, L) and the occupied alveolus volume fraction, in black (M, N, O). Thin and bright lines represent single simulations, 10 single simulations are shown as example for each set. Thick and dark lines are the mean value of 10000 simulations. Simulations were 200 days long, but only the first 140 are shown.

Single alveolus model results are computed eliminating diffusive (blue) parts of Eqs (1)–(10). Exploring different set of parameters and diverse initial conditions we see that there are two possible final states as it can be seen in Fig 3. (1) Proliferative scenario: bacilli population is eliminated by a flux of macrophages that are activated by T cells. Temporally, part of the alveolus is filled by macrophages, however, finally the number of macrophages returns to 1. Some of the space remains occupied by dead cells. (2) Exudative scenario: bacilli persevere and are able to kill all macrophages that try to contain the infection. Most of the present cells in this scenario are neutrophils thus favouring extracellular growth of bacilli. At the end, all bacilli are extracellular due to macrophages lysis and alevolus is occupied by dead cells of macrophages, neutrophils and T cells.

Under certain values of the parameters, bistability of both states is observed and the fluctuations determine the final state. According to these different simulation possibilities we can define three zones of parameters: Proliferative zone where all simulations end in a proliferative scenario, bistable region where a simulation can end in exudative or proliferative scenario depending on the particular realization, and exudative zone where all simulations end in exudative scenario. For each set of parameters we can compute the probability to end in exudative state, *p_exu_*, or proliferative scenario, *p_pro_*. Note that *p_exu_* = 1 − *p_pro_*.

#### Activation rate of macrophages determines transition between proliferative and exudative scenarios

In Fig 3, a sample of simulations with three different set of parameters are shown, one set for each defined zone (proliferative, bistable and exudative). The parameter used to explore different parameters zone is macrophage activation rate (*ξ*). Different values used were: proliferative (*ξ* = 0.5 day^-1^), bistable (*ξ* = 0.15 day^-1^) and exudative (*ξ* = 0.01 day^-1^). For the set of parameters of the bistable zone, the probability to end in exudative scenario is *p*_*exu*_ = 0.22. This probability is computed using the results of 10000 simulations till day 200.

Initial behaviour is very similar for all sets of parameters, inflammatory response is triggered by first macrophages lysis that takes places 6.4 ± 2.3 days after initial infection and its fully awaken around day 16th. Firsts T cells arrive at the alveolus at day 14.2 (1 T cell), and around day 17th there are around 25. At day 16.7 ± 2.8, the first activated macrophage is observed, after 19th day more than 10 macrophages are active.

#### Transition to exudative scenario depends on bacilli growth and T cells and macrophage activity

**Fig 4:**
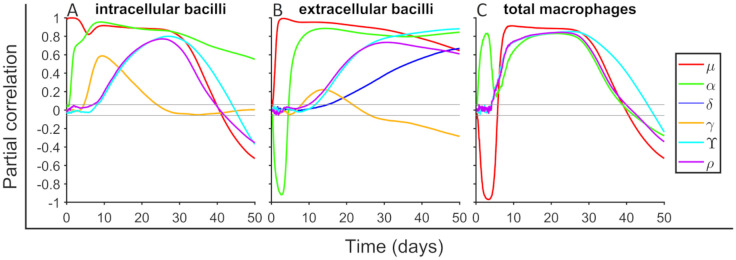
Sensitivity analysis for intracellular bacilli, extracellular bacilli and total macrophages. Partial Rank Correlation Coefficient (PRCC) is computed between a total of six input parameters (see legend) and outcome variables. A total of 1500 parameters sets are used for exploring the parameter space defined in Table 1 using Latin Hypercube Sample technique. A total of 14 parameters are explored, the ones that are not shown it is because their PRCC values is smaller than 0.2. Gray horizontal lines are marked at ±0.043 to mark significance threshold for PRCC values using a significance level of 0.05. PRCC are computed in each time step (dt = 0.1 day). For each set of parameters a total of 10000 simulations are done.

We perform a sensitivity analysis to determine the input parameters effect in outcome variables. We employ the methodology described in [40]. Parameters are sampled using a Latin Hypercube Sample technique [41] between the values specified in Table 1. We explore a total of 14 parameters, which are those that do not have a well defined value. In most of the cases, the exploration ranges are between half and double of their baseline value, but in some parameters of higher interest exploration ranges are extended. We use 1500 points to perform the exploration. Partial Rank Correlation Coefficient (PRCC) is computed between input parameters and outcome variables along time. In Fig 4 we show the correlation between six parameters (*μ*, *α*, *δ*, *γ*, *Υ* and *ρ*) and the outcomes intracellular bacilli (*b*_*I*_), extracellular bacilli (*b*_*E*_), and total macrophages (*m*). Parameters *κ*, *ν*, *ξ*, λ_*b*_, λ_*m*_, λ_*T*_, *τ*_*m*_ and *t*_*n*_ are not shown in Fig 4 because they do not present correlations bigger than 0.2 with these outcome variables. It can be seen that as expected parameter *μ* has a positive correlation with the number of bacilli, long term correlations decrease because alveolus is filled. Initially *μ* reduces macrophages quantity due to bacilli growing inside, but later this effect triggers inflammatory response and macrophages numbers increase. Parameter *α* contributes to increase bacilli quantities because more bacilli can duplicate inside a macrophage, these also trigger inflammatory response and more macrophages enter to the alveoli. Parameter *δ* increases the number of extracellular bacilli because more bacilli can duplicate over a neutrophil. Parameters *Υ* and *ρ* have a very similar effect because both are related with inflammatory response (*Υ* increases the entering flux and *ρ* activates faster the inflammatory response). This accelerates the process and more bacilli and macrophages are seen before. Parameter *γ* is important to determine how fast are bacilli engulfed and its effect is mainly seen after first macrophage lysis.

**Fig 5:**
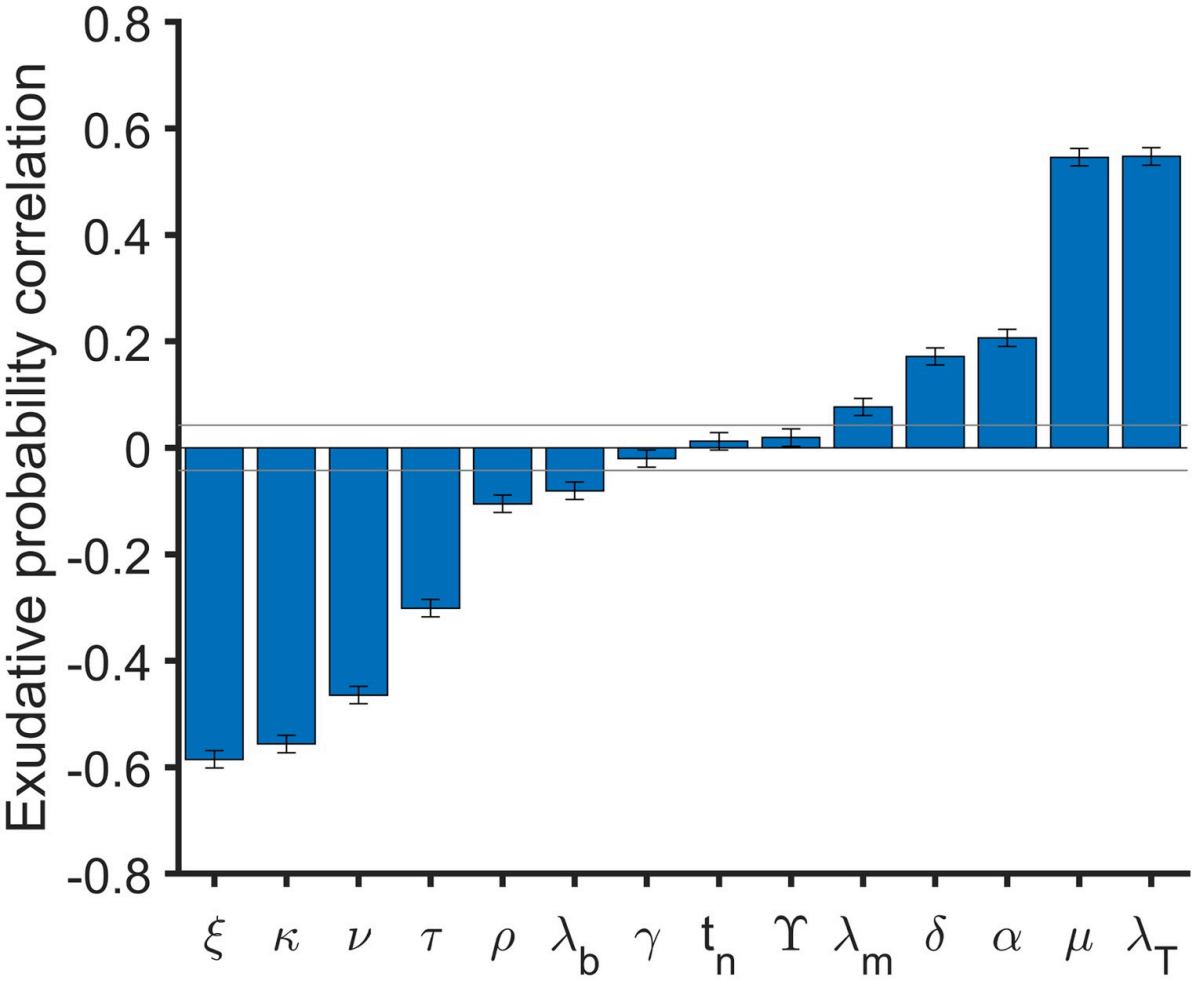
Partial Rank Correlation Coefficient (PRCC) between input parameters and exudative probability. PRCC between *ξ*, *κ*, *ν*, λ_*b*_, *γ*, *t*_*n*_, *Υ*, λ_*m*_, *δ*, *α*, *τ*_*m*_ and λ_*T*_ and exudative probability are shown in blue bars. Infection probability is computed at time = 200 day as the presence or not of bacilli (*b*_*I*_ + *b*_*E*_). Gray horizontal lines are marked at ±0.043 to mark significance threshold for PRCC values using a significance level of 0.05. A total of 1500 parameters sets are used to explore the parameter space defined in Table 1 using Latin Hypercube Sample technique. For each set of parameters a total of 10000 simulations are done. Error bars are computed as the standard error of Spearman’s correlation coefficient.

As seen in Fig 3, the set of parameters determines final state probabilities. We compute PRCC between 14 input parameters used in sensitivity analysis and the probability to end in exudative scenario. In Fig 5 PRCC final values are shown. Increasing T cells death rate (λ_*T*_) and macrophages growing rate (*μ*) or reducing macrophages activation (*ξ*), T cells recuitment (*κ*), activated macrophages killing rate (*ν*) and activated macrophages lifespan (*τ*_*m*_) increase the probability to end in exudative scenario. Therefore, parameter *ξ* is not the unique parameter that determines the zone where a desired set of parameters belong.

### Model of secondary lobule

#### Fibroblast encapsulation stops the expansion of the lesion in the model

**Fig 6:**
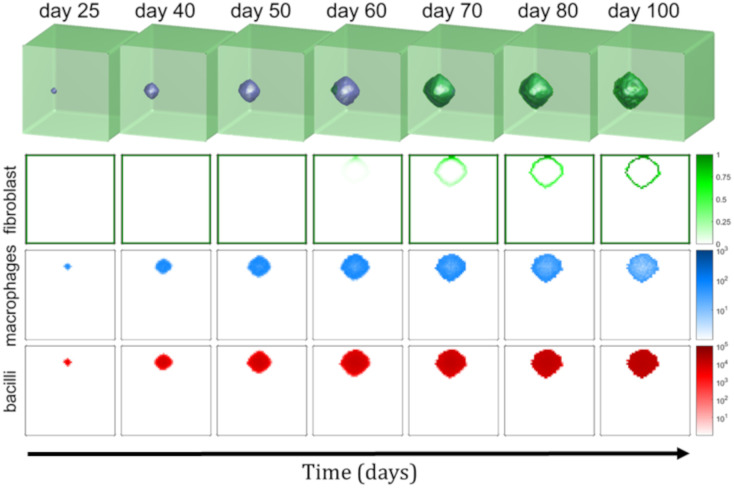
Evolution of 3D model. Evolution of the secondary lobule model in a cubic 50 × 50 × 50 alveoli grid. The first row is the 3D representation of the secondary lobule. Fibroblasts are shown in green and the occupied space (macrophages, T cells, dead cells, bacilli…) is in blue. Second, third and fourth rows represents a slice (z plane at the height of initial infection). Concentration of fibroblasts in shown in green (second row), the total number of macrophages (uninfected, infected and activated) in logarithmic scale in blue (third row) and, finally, the total number of bacilli (intracellular and extracellular) in logarithmic scale in red (fourth row). These quantities and representations can be observed at different days after initial infection, each column represents a different time of simulation.

Initially all alveoli present one uninfected macrophages except a certain alveolus that has an infected macrophage with a bacillus inside. The resulting evolution of the infection can be observed in Fig 6. We show the total bacilli amount (*b*), total macrophages amount (*m*) and fibroblasts concentration (*f*). In supplementary material there is a video with lesions growth and encapsulation process.

Initial infection starts with an infected macrophage and an intracellular bacillus that duplicates. Intracellular bacilli duplicate until lysis of first macrophage; then, approximately *α* extracellular bacilli are released and diffuse to nearby alveoli. Infection continues as seen in Fig 3 (exudative set) but extending to surrounding alveoli. It forms an spherical lesion with a radius that grows exponentially. This unstoppable process continues until lesion makes contact with secondary lobule membrane (*septae*), which occurs around day 51 as seen in Fig 6, where baseline values are used. This contact triggers the encapsulation process. Fibroblasts contain lesions growth and an encapsulated lesion is formed (day 73). Encapsulation process finishes around day 126 and no diffusion is observed after this time. The shape of the lesion is spherical because encapsulation process is faster than lesion growth. If encapsulation is slower (it can be seen with a different set of parameters), the shape of the lesion is oval. In fact, if fibroblasts chemo-taxis is slow enough, lesions may occupy the whole secondary lobule and encapsulation is not formed.

#### Encapsulation time and volume of the lesions depends on fibroblast spreading

An uncertainty analysis is performed following the same procedure as in the single alveolus model. A total of 100 simulations are run, exploring 16 input parameters (Table 1). Diffusion coefficients are also varied using two parameters, D and DF. Values of diffusion coefficients are related following:

$$\begin{array}{c} D_{m}=D_{n}=D_{T}=10\cdot D_{b}=\frac{\chi_{f}}{\text{DF}}=D. \end{array}$$ (24)

**Fig 7:**
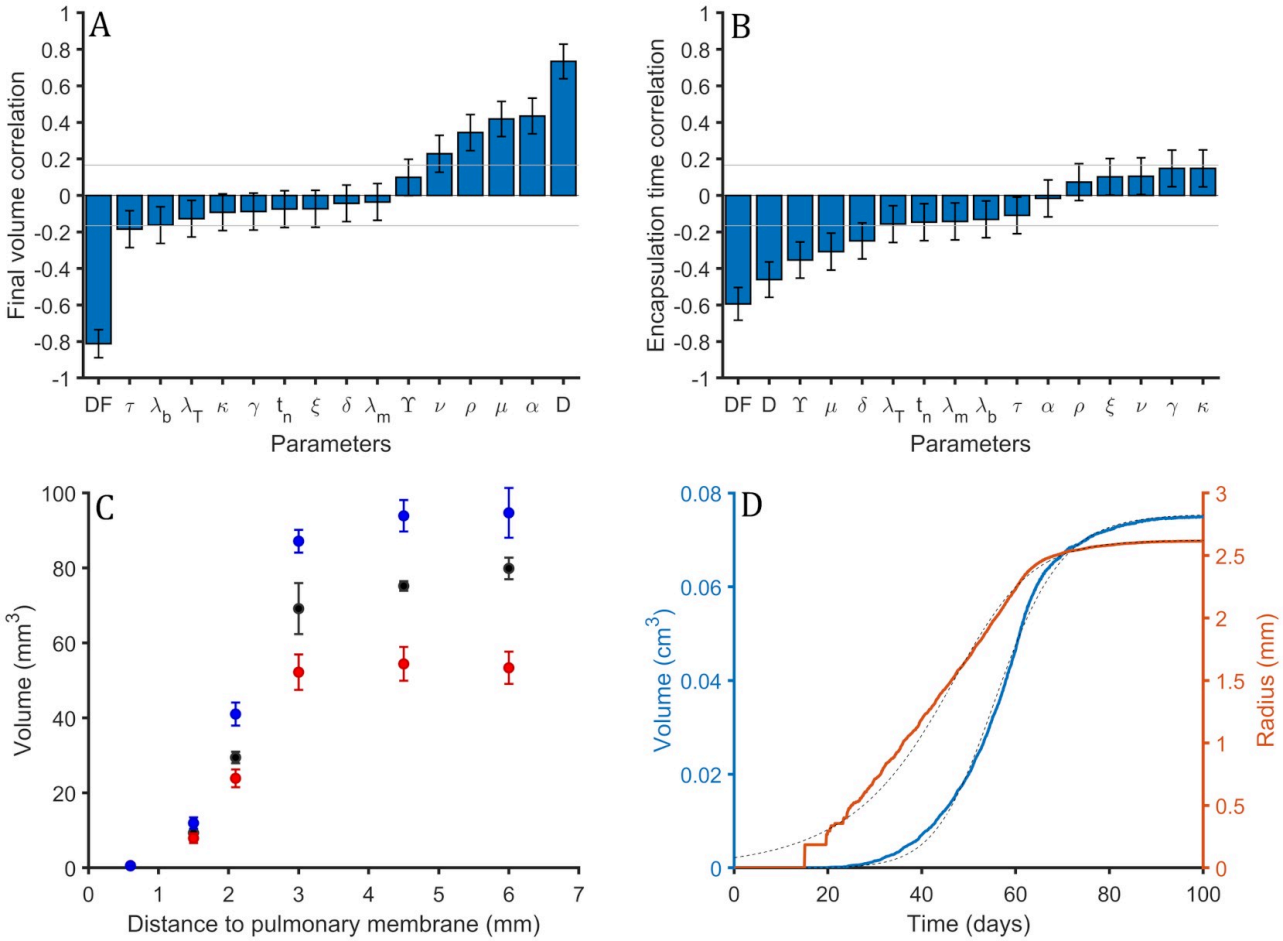
Sensitivity analysis of 3D model. (A) Partial Rank Correlation Coefficient (PRCC) values between volume of the final lesion and explored input parameters of the model. 100 sets of input parameters are used. Gray horizontal lines are marked at ±0.166 to mark the significance threshold for PRCC values using a significance level of 0.05. Error bars are computed as the standard error of Spearman’s correlation coefficient. (B) PRCC values between encapsulation time and input parameters. 100 sets of input parameters are used. Gray horizontal lines are marked at ±0.165 to mark the significance threshold for PRCC values using a significance level of 0.05. Error bars are computed as the standard error of Spearman’s correlation coefficient. (C) Distance to the nearest pleura against the volume of the resulting lesion. Each point is the mean value and standard deviation for three different simulations. Different values of D are explored. In black, baseline value, in red, baseline value divided by 2 and, in blue, baseline value multiplied by 2. (D) Volume, in blue, and radius, in orange, of the lesion using baseline values. Dotted black line is generalized logistic second degree curve approximation defined by Eqs 25–27.

Parameter D is varied between the double and its half of its baseline value and DF is explored between 0.5 and 4 times its baseline value. PRCC are computed between input parameters and outcome variables. Total amount of intracellular and extracellular bacilli, and macrophages are similar than the values seen in the single alveolus model, see Fig 4. In Fig 7A we observe PRCC values between volume of the lesions and the explored parameters. Lesions volume is determined by two initial parameters D, diffusion of the elements, related with lesions growth, and DF, fibroblasts diffusion, related with encapsulation velocity. If the lesions grow faster it is more difficult to encapsulate them and final lesions are bigger. In fact, the faster the encapsulation speed, the smaller the lesion. This relation is observed in Fig 7B. Size of the lesions is also determined (less influence than D and DF) by *α*, *μ* and *ρ*. These 3 parameters increase bacillary load, as shown in Fig 4. This causes a higher diffusion of bacilli and macrophages, thus a bigger lesion.

In Fig 7B there is PRCC between encapsulation time and input parameters. Encapsulation time is defined as the elapsed time between first time that fibroblast diffusion is non zero and the time where it becomes zero again. Parameter DF reduces encapsulation time because it increases fibroblasts diffusion and, as seen in Fig 7A, parameter D increases final lesions volume.

We have observed that bigger lesions have a larger encapsulation time, then we would expect that D increases encapsulation time. However, in Fig 7B it is seen that, contra-intuitively, it decreases. This is due to the fact that increasing D also increases fibroblasts diffusion but, in fact, as the mean value of DF is higher than 1 (it is 2.25) the increase in fibroblasts diffusion is amplified. Parameters DF and D are not the only parameters that determine lesions final volume.

Note that Fig 7 error bars are bigger than the ones observed previously in Fig 5, due to the low number of realizations done in the secondary lobule model, because it is more computationally demanding.

#### Distance to the nearest pulmonary membrane strongly determines final volume of the lesion

Different simulations are carried out with different initial infection positions. As cubic secondary lobules are considered, the distance to membranes can be computed from three different axis (x, y and z). The initial infection is considered in the middle of the z axis. Then, the considered distances are 7.5 mm for each one of the sides. The distance to y membrane is fixed at 3 mm for the closest side and 12 mm for the other side. The distance to x membrane is varied for the different simulations. The considered distances are: 0.6 mm, 1.5 mm, 2.1 mm, 3.0 mm, 4.5 mm and 6.0 mm. For each different distance, three simulations are run. All simulations are repeated considering a doubled baseline value for D and using the half of its value. In Fig 7C the volume of the lesion as a function of the distance to the nearest x membrane for the three different values of D can be seen.

Two different behaviours can be distinguished. When the distance is smaller than 3 mm, a clear proportionality between distance and volume is observed. The bigger the distance the bigger the volume. As small data is available, it can not be distinguished between exponential growth or a polynomial growth of a certain power. It is expected that volume is proportional to the cube of the distance to the nearest membrane.

When the distance is bigger than 3 mm, this behaviour changes. This is due to that the nearest distance thought x axis (that is the plotted one) is not the small one because the nearest y membrane is at 3 mm. It can be seen that the volume does not increase now with the distance. Then it can be seen a clear dependence between the volume of the lesion and the distance to the closest membrane.

In Fig 7C it is seen that the volume of the lesions increase with the diffusion (bigger values of D parameter). In blue simulations with the double of D value, in black the baseline value and in red half of the baseline value. All the colors follow the same shape. This result is also seen in Fig 7A.

#### Radius of the lesion can be fitted with a logistic equation

In Fig 7D lesions growth is observed (volume in blue and radius in orange) for a single secondary lobule simulation using as initial infection point an alveolus that is at 2.7 mm of the closest membrane. This growth can be approximated by a generalized logistic function [42] of second degree for the radius:

$$\begin{array}{c} \frac{d}{\text{dt}}r\left(t\right)=v\cdot r\left(t\right)\cdot (1-{\left(\frac{r(t)}{r_{max}}\right)}^{2}). \end{array}$$ (25)

That can be solved imposing that there is a minimum radius (r_min_) at a desired time (t_min_):

$$\begin{array}{c} r\left(t\right)=\frac{r_{max}r_{min}}{\sqrt{r_{min}^{2}+\left(r_{max}^{2}-r_{min}^{2}\right)\cdot exp(-2v(t-t_{min}))}}. \end{array}$$ (26)

Catala et al [18] adjusted an agent-based model based on experiment observations of minipigs. A logistic growth was adjusted using the following parameters: r_max_ = 2.62 mm, v = 0.1324 day^-1^, r_min_ = 0.0207 mm and t_min_ = 14 days. Imposing t_min_ = 14 days to obtain comparable results we can adjust the logistic as:

$$\begin{array}{c} \begin{array}{c} r_{max}=2.62\;\text{mm},\\ v=0.1324\;{\text{day}}^{-1},\\ r_{min}=0.0207\;\text{mm}. \end{array} \end{array}$$ (27)

Comparing both models it can be seen that in this model (human) lesions are assumed to be bigger but to grow slower than minipig ones.

## Discussion

Single alveolus model is useful to study macrophages-bacilli interaction observing two possible outcomes (three zones of parameters can be distinguished). In the bistability both final states and the probabilities of finishing in each one can be computed. The parameters that reduce bacillary load and exudative scenario probability are identified (↓*μ*, ↓λ_*T*_, ↑*ξ*, ↑*κ*, ↑*ν* and ↑*τ*_*m*_) these parameters can be related with biological processes. Then, biological processes that reduce bacillary load and exudative scenario probability are a decrease of bacilli reproduction, an increase in T cells lifespan, an increase in activated macrophages lifespan, a faster activated macrophages killing rate, a faster T cells recruitment speed or a faster macrophages activation rate.

Using baseline parameters infection can not be controlled and bacilli resist inflammatory and immune response. The encapsulation process is needed (secondary lobule model) to stop bacilli proliferation. This process controls lesion growth when immune response is not enough as seen in [7] and in the single alveolus model. The bacilli growth is not stopped but the affected alveoli are controlled not allowing to infect surrounding ones.

The modeling of such encapsulation process is closely related with the description of the growth of the granuloma. There have been several attempts to model such growing basically based in agent based models for the cells and their motion among aveoulus of the same secondary lobule [13, 17] and in the other hand reaction diffusion models for the number of cells inside the secondary lobule [43]. Our model also describes the growing of the granuloma by the use of reaction-diffusion equations, however, we incorporate in the model the saturation of such growing due to the interaction with the interlobular connective tissue by the description of the dynamics of the fibroblast.

As consequence of the approach, lesions size is affected by its position on the lung, in particular, the distance from its focus to the nearest pulmonary membrane. Other two factors that determine its size is the small diffusion velocity of the elements that is reduced by fibroblasts, collagen and other molecules present in alveoli (encapsulation velocity) and the facility that infected macrophages can diffuse to surrounding alveoli (lesion growth). Secondary lobule size and shape is important to control lesions before, therefore to control infection and not evolve to ATB. We have seen that smaller secondary lobule (smaller lungs) present smaller lesions and TB is better controlled. This could be a reason to justify the lower incidence in children than in adults observed in [44].

Volume and radius evolution of the lesions can be successfully adjusted to a generalized logistic equation as predicted by the bubble model employed in some previous empirical approaches for the growth of the granuloma [17, 18].

## Further work

Next step is to build a computational lung that can reproduce TB disease that includes: bronchial tree structure [18] with a secondary lobules structure that includes pulmonary membranes and the model described before.

The details of the model can be refined including some intermediate steps that were simplified in the processes of macrophages clearance or immune activation. To study post lesion formation scenario it must be considered that when encapsulation is completed foamy macrophages can not travel outside the lesion, then process 14 (intracellular bacilli drainage) is inhibited in that lesion.

Another important point that may be taken into account is how ventilation and blood flow affect the different parts of the lung. In particular, secondary pulmonary lobules are in different conditions depending on the lung part: ventilation, blood flow, shape and size are different. These conditions may change some parameters and end in different lesions outcome.

Lesions much bigger than a single secondary lobule are obtained in active tuberculosis. In order to model the break of the pulmonary membrane, some other mechanical processes could be considered.

## Supporting information

S1 Video Encapsulation of a tuberculosis lesion in a 3D polygon secondary lobule. In green fibroblast that surrounds secondary lobule and encapsulates the lesion. In blue cells that form the lesion (macrophages, neutrophils, T cells…). Lesion grows till it contacts with interlobular connective tissue and it is encapsulated.
(AVI)
